# Supplementary material for: Molecular evolution of DNMT1 in vertebrates: Duplications in marsupials followed by positive selection
Source: PLoS One. 2018 Apr 5;13(4):e0195162. doi: 10.1371/journal.pone.0195162 (PMC5886458; doi:10.1371/journal.pone.0195162)
Supplement: S1 Fig — The human sequence corresponds to the Dnmt1s isoform. Dashes represent alignment gaps or missing regions. Stretches of “X” symbols represent unsequenced regions. Single “X” symbols represent incomplete codons (e.g., due to frameshift mutations). (PDF) [file pone.0195162.s001.pdf]

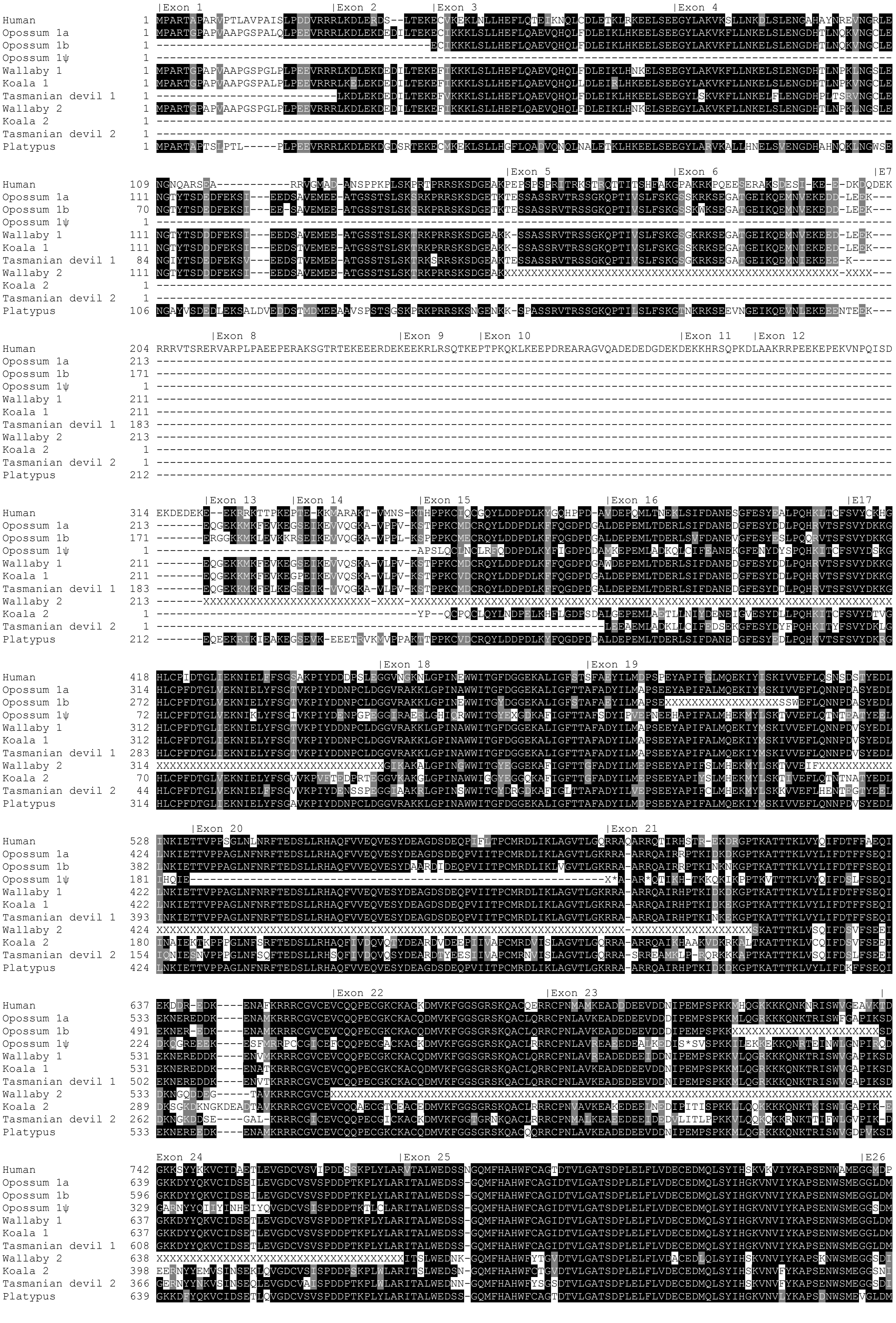

Human
Opossum 1a
Opossum 1b
Opossum 1ψ
Wallaby 1
Koala 1
Tasmanian devil 1
Wallaby 2
Koala 2
Tasmanian devil 2
Platypus
|Exon 1
|Exon 2
|Exon 3
|Exon 4
|Exon 5
|Exon 6
|E7
|Exon 8
|Exon 9
|Exon 10
|Exon 11
|Exon 12
|Exon 13
|Exon 14
|Exon 15
|Exon 16
|E17
|Exon 18
|Exon 19
|Exon 20
|Exon 21
|Exon 22
|Exon 23
Exon 24
|Exon 25
|E26
