# Supplementary material for: Molecular evolution of DNMT1 in vertebrates: Duplications in marsupials followed by positive selection
Source: PLoS One. 2018 Apr 5;13(4):e0195162. doi: 10.1371/journal.pone.0195162 (PMC5886458; doi:10.1371/journal.pone.0195162)
Supplement: S2 Fig — The human sequence corresponds to the Dnmt1s isoform. Dashes represent alignment gaps or missing regions. Stretches of “X” symbols represent unsequenced regions. Single “X” symbols represent incomplete codons (e.g., due to frameshift mutations). (PDF) [file pone.0195162.s002.pdf]

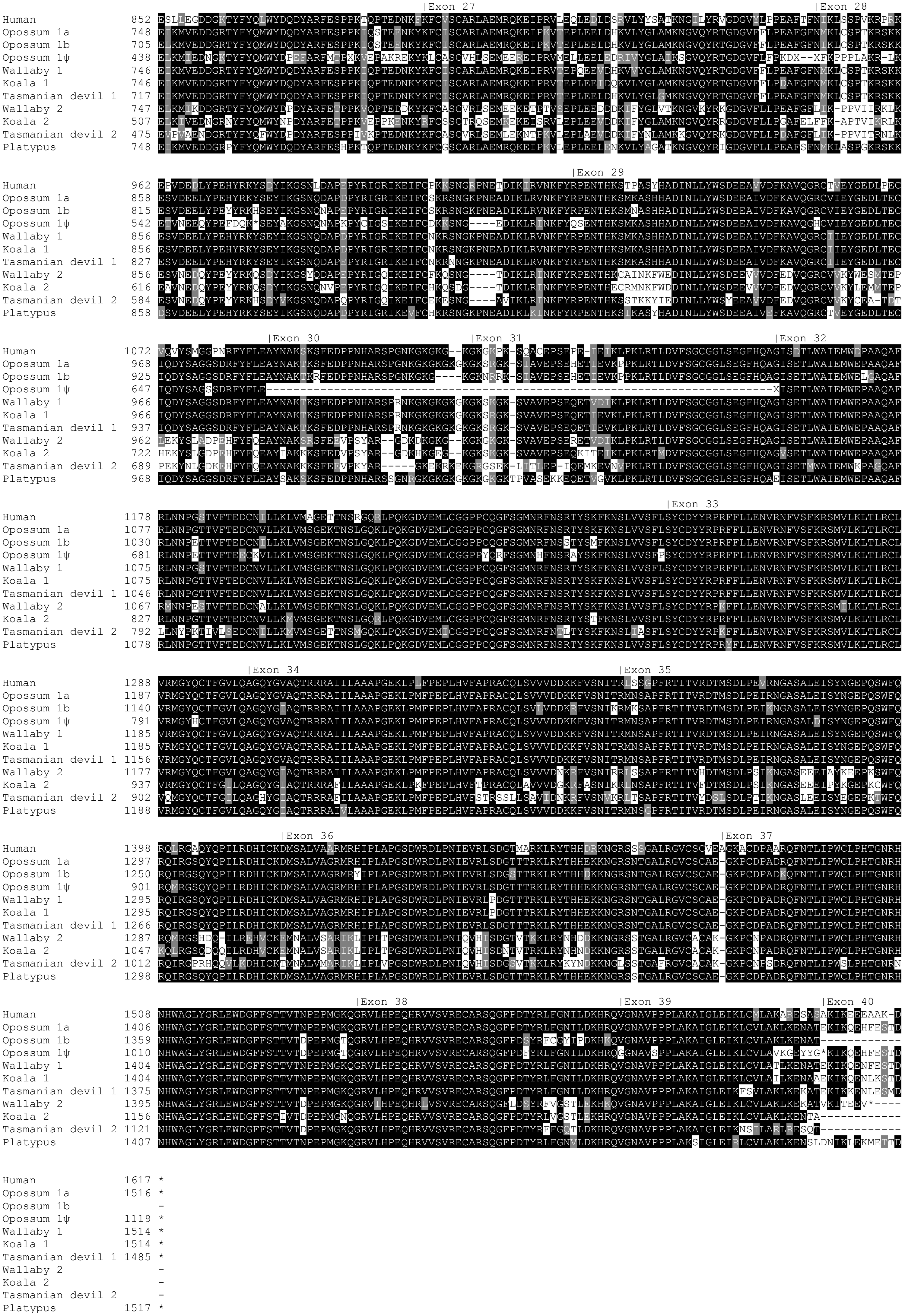

|Exon 27
|Exon 28
|Exon 29
|Exon 30
|Exon 31
|Exon 32
|Exon 33
|Exon 34
|Exon 35
|Exon 36
|Exon 37
|Exon 38
|Exon 39
|Exon 40
