# Supplementary figures and images for: Molecular evolution of DNMT1 in vertebrates: Duplications in marsupials followed by positive selection
Source: PLoS One. 2018 Apr 5;13(4):e0195162. doi: 10.1371/journal.pone.0195162 (PMC5886458; doi:10.1371/journal.pone.0195162)

# Tasmanian devil scaffold GL841374.1

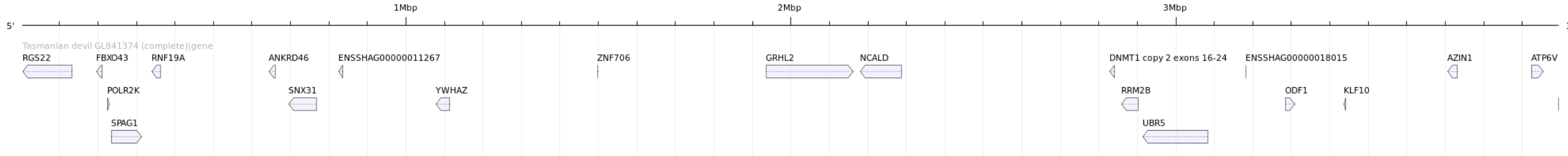

Supplement: S3 Fig — The scaffold contains part of DNMT1 copy 2. For unnamed non-human genes, the name of the human ortholog (according to Ensembl’s annotations) is shown. Gene coordinates were extracted from the Ensembl database, except for DNMT1 copy 2, for which we used our manually refined annotations. Genome visualizations were generated using GenomeTools [46]. (PDF) [file pone.0195162.s003.pdf]
